# Prognostic Impact of PCK1 Protein Kinase Activity-Dependent Nuclear SREBP1 Activation in Non-Small-Cell Lung Carcinoma

**DOI:** 10.3389/fonc.2021.561247

**Published:** 2021-03-26

**Authors:** Fei Shao, Xueli Bian, Juhong Wang, Daqian Xu, Wei Guo, Hongfei Jiang, Gaoxiang Zhao, Lei Zhu, Shuai Wang, Dongming Xing, Yibo Gao, Jie He, Zhimin Lu

**Affiliations:** ^1^ Cancer Institute of the Affiliated Hospital of Qingdao University, Qingdao Cancer Institute, Qingdao, China; ^2^ Department of Thoracic Surgery, National Cancer Center/National Clinical Research Center for Cancer/Cancer Hospital, Chinese Academy of Medical Sciences and Peking Union Medical College, Beijing, China; ^3^ Zhejiang Provincial Key Laboratory of Pancreatic Disease of the First Affiliated Hospital, Institute of Translational Medicine, Zhejiang University School of Medicine, Hangzhou, China; ^4^ School of Life Sciences, Tsinghua University, Beijing, China; ^5^ State Key Laboratory of Molecular Oncology, National Cancer Center/National Clinical Research Center for Cancer/Cancer Hospital, Chinese Academy of Medical Sciences and Peking Union Medical College, Beijing, China

**Keywords:** PCK1, INSIG1/2, SREBP1, phosphorylation, lipogenesis, prognosis, non-small-cell lung carcinoma, immunohistochemistry

## Abstract

Metabolic enzymes can perform non-metabolic functions and play critical roles in the regulation of a variety of important cellular activities. Phosphoenolpyruvate carboxykinase 1 (PCK1), a gluconeogenesis enzyme, was recently identified as an AKT-regulated protein kinase that phosphorylates INSIG1/2 to promote nuclear SREBP1-dependent lipogenesis. However, the relationship of this regulation with the progression of non-small-cell lung carcinoma (NSCLC) is unclear. Here, we demonstrate that epidermal growth factor receptor (EGFR) activation induces AKT-dependent PCK1 pS90, PCK1-mediated INSIG1 pS207/INSIG2 pS151, and nuclear SREBP1 accumulation in NSCLC cells. In addition, the expression levels of AKT pS473, PCK1 pS90, INSIG1 pS207/INSIG2 pS151, and nuclear SREBP1 are higher in 451 analyzed human NSCLC specimens than in their adjacent normal tissues and positively correlated with each other in the tumor specimens. Furthermore, the expression levels of PCK1 pS90, INSIG1 pS207/INSIG2 pS151, and nuclear SREBP1 are associated with TNM stage and progression in NSCLC. Importantly, levels of PCK1 pS90 or INSIG1 pS207/INSIG2 pS151 are positively correlated with poor prognosis in NSCLC patients, and the combined expression value of the PCK1 and INSIG1/2 phosphorylation has a better prognostic value than that of each individual protein phosphorylation value and is an independent prognostic marker for NSCLC. These findings reveal the role of PCK1-mediated nuclear SREBP1 activation in NSCLC progression and highlight the potential to target the protein kinase activity of PCK1 for the diagnosis and treatment of human NSCLC.

## Introduction

Lung cancer is a leading cause of cancer-related death worldwide (1). Non-small-cell lung carcinoma (NSCLC) is the most common type of lung cancer, accounting for approximately 85% of all cases (2). Its prognosis remains poor because of the high rates of metastasis, recurrence, and drug resistance (3). Therefore, identification of novel biomarkers is vital to improve the diagnosis and treatment of NSCLC patients.

Metabolism reprogramming is an emerging hallmark of cancer biology (4–6). For instance, cancer cells acquire energy mainly through upregulated glycolysis but not oxidative phosphorylation. This characteristic of cancer cells is known as the Warburg effect. As the reverse process of glycolysis, gluconeogenesis converts non-glycolytic precursors to glucose. Phosphoenolpyruvate (PEP) carboxykinase 1 (PCK1), the rate-limiting enzyme of gluconeogenesis, converts oxaloacetate and GTP into PEP and CO_2_ and is regarded as a tumor suppressor because of its inhibitory effect on glycolysis (7). Our recent studies demonstrated that AKT activation in response to activation of growth factor receptors, including insulin-like growth factor 1 receptor (IGF1R), epidermal growth factor receptor (EGFR), and platelet-derived growth factor receptor (PDGFR), and active K-RAS mutations, results in the binding of AKT to PCK1 and AKT-mediated phosphorylation of PCK1 at S90. Phosphorylated PCK1 translocates to the endoplasmic reticulum (ER) and is associated with INSIG1/2. Importantly, PCK1 acts as a protein kinase by using GTP as a phosphate donor and phosphorylates INSIG1 S207 and INSIG2 S151 (INSIG1 pS207/INSIG2 pS151), which are in cytosolic loop 2 of INSIG1/2. This phosphorylation reduces the binding of sterol to INSIG1/2, leading to disruption of the INSIG-SCAP interaction, translocation of the SCAP-sterol regulatory element-binding protein 1 (SREBP1) complex to the Golgi apparatus, cleavage of SREBP1, and nuclear accumulation and activation of nuclear SREBP1. As a result, the transcription of the genes for lipogenesis is activated. In addition, activated nuclear SREBP1 induces its own gene transcription in a positive feedback manner. Blockade of AKT-mediated phosphorylation of PCK1 and PCK1-mediated phosphorylation of INSIG1/2 inhibits lipid synthesis, hepatocellular carcinoma (HCC) cell proliferation, and tumor formation in mice (8, 9). These findings underscore the significance of the newly identified protein kinase activity of gluconeogenesis enzyme PCK1 in nuclear SREBP1 activation, lipogenesis, and HCC development (8). Although the role of PCK1-dependent lipogenesis in HCC cell proliferation was revealed, whether AKT-phosphorylated PCK1 pS90, INSIG1 pS207/INSIG2 pS151, or nuclear SREBP1 expression is associated with progression and prognosis in NSCLC patients is unknown.

In this study, we demonstrated that EGFR activation induces AKT-dependent PCK1 pS90, PCK1-mediated INSIG1 pS207/INSIG2 pS151, and nuclear SREBP1 accumulation in NSCLC cells. In addition, the levels of AKT pS473, AKT-dependent PCK1 pS90, PCK1-mediated INSIG1 pS207/INSIG2 pS151, and nuclear SREBP1 expression were higher in human NSCLC specimens than in their adjacent normal tissues. Importantly, AKT-dependent and PCK1-mediated INSIG1/2 phosphorylation and nuclear SREBP1 expression were associated with progression and poor prognosis in NSCLC.

## Materials and Methods

### Cell Lines and Cell Culture Conditions

H1395, H226, A549, H358, H460, H1299, H1993, and H322M NSCLC cells were from ATCC. The cells were maintained in Dulbecco’s modified Eagle’s medium (DMEM) supplemented with 10% fetal bovine serum (FBS), 1,000 U ml^−1^ penicillin, and 100 μg ml^−1^ streptomycin. Before EGF (100 ng ml^−1^) treatment, the cells were serum-starved for 16 h. Cell lines were authenticated by short tandem repeat profiling and were routinely tested for mycoplasma contamination at Qingdao Cancer Institute. Cells were plated at a density of 4 × 10^5^ per 60-mm dish 18 h before transfection. The transfection procedure was performed, as previously described (10).

### DNA Construction and Mutagenesis

PCR-amplified human wild-type (WT) PCK1, INSIG1, and INSIG2 were cloned into pcDNA3.1/hygro(+)-Flag, pCDH-CMV-MCSEF1-Puro-SFB or pET32a vectors. pcDNA3.1 rPCK1 S90A, rINSIG1 S207A, rINSIG2 S151A were mutated using a QuickChange site-directed mutagenesis kit (Stratagene). pGIPZ shRNA was constructed *via* ligation of an oligonucleotide targeting human PCK1 into an XhoI/MluI-digested pGIPZ vector. The following pGIPZ shRNA target sequences were used: control shRNA oligonucleotide, 5’-GCTTCTAACACCGGAGGTCTT-3’; *PCK1* shRNA oligonucleotide, 5’-TGTGCGTCAAACTTCATCC-3’; *INSIG1* shRNA oligonucleotides, 5’-TAATGGTGTCTATCAGTATAC-3’ and 5’-GGAACATAGGACGACAGTTA-3’; *INSIG2* shRNA oligonucleotides, 5’-CATCTAGGAGAACCTCATAAA-3’ and 5’- CTTCAGCTGTGATTGGGTT-3’.

### Immunoprecipitation and Immunoblotting Analysis

The extraction of proteins using a modified buffer from cultured cells was followed by immunoprecipitation and immunoblotting using corresponding antibodies, as described previously (11).

### Mouse Studies

One million H1395 cells with expression of rPCK1, rPCK1 S90A, rINSIG1/INSIG2 or rINSIG1 S207A/rINSIG2 S151 double mutants were collected in 20 μl DMEM with 33% matrigel and subcutaneously injected into 6-week-old female BALB/c athymic nude mice. The injection was performed, as described previously (12). Six mice per group in each experiment were used. Mice were euthanized 28 days after injection. The tumors were dissected and then fixed in 4% formaldehyde. The mice were treated in accordance with relevant institutional guidelines and regulations. The use of the mice was approved by the Institutional Review Board and the Institutional Animal Care and Use Committee (IACUC) of Qingdao Cancer Institute, China. Mice arriving in the animal facility were randomly put into cages with five mice each. No statistical methods were used to predetermine sample size.

### Patients and Tissue Samples

We retrospectively collected surgically resected, formalin-fixed, paraffin-embedded NSCLC tissue samples from the biobank of the Affiliated Hospital of Qingdao University (Qingdao, China) and the National Cancer Center/National Clinical Research Center for Cancer/Cancer Hospital, Chinese Academy of Medical Sciences and Peking Union Medical College (Beijing, China). Tissue samples from 450 treatment-naïve patients who underwent surgery for pathologically diagnosed cancer between 2002 and 2013 were selected as an independent cohort, including 306 cases of lung adenocarcinoma (LUAD) (with 302 paired normal specimens) and 145 cases of lung squamous cell carcinoma (LUSC) (with 145 paired normal specimens). The patient cohort is not population-based, but consists of consecutive patients.

We obtained clinical data by reviewing the patients’ medical histories. Pathological staging was assessed by the 8^th^ edition of the American Joint Committee on Cancer/Union for International Cancer Control TNM classification system (13).

### Tissue Microarray Construction

Anti-AKT pS473 (#4060) rabbit antibody (for IHC) and a rabbit monoclonal antibody recognizing IgG were purchased from Cell Signaling Technology (Danvers, MA). Rabbit antibodies that recognize PCK1 pS90, INSIG1 pS207 and INSIG2 pS151 were obtained from Signalway Biotechnology (Pearland, TX). Nuclear SREBP1 antibody (2A4) (NB100-2215) (for IHC analyses) was purchased from Novus (Littleton, CO). The specificities of these antibodies were previously validated (8). Formalin-fixed and paraffin-embedded tissues were obtained by surgical resection and stained with Mayer’s hematoxylin and eosin (H&E; Biogenex Laboratories, San Ramon, CA).

Tumor samples from 451 cancer cases with 447 paired normal tissues were subjected to tissue microarray (TMA). Employing an automated tissue array instrument (Minicore^®^ 3, Alphelys, Plaisir, France), cancer tissue (diameter at 2 mm, selected by a pathologist) from each specimen was extracted and fixed into a paraffin block. After quality control, the TMA blocks were sectioned into slides for immunohistochemistry analysis.

### Immunohistochemistry and Evaluation

Immunohistochemistry analyses were performed according to a previous publication (14). After deparaffinization, rehydration, and antigen retrieval, TMA slides were incubated with primary rabbit anti-human AKT pS473 (dilution 1:200), primary rabbit anti-human phospho-PCK1 S90 (dilution 1:200), primary rabbit anti-human INSIG1 pS207 and INSIG2 pS151 (dilution 1:500), primary rabbit anti-human SREBP1 (dilution 1:100) or nonspecific IgG (as a negative control) overnight at 4°C. The slides were then incubated with anti-rabbit secondary antibody (Cell Signaling Technology; #8114), followed by chromogen diaminobenzidine (DAB) (Cell Signaling Technology) and hematoxylin staining. We quantitatively scored the tissue slides under a microscope according to the percentage of positive cells and staining intensity. We assigned the following proportion scores: 0, 0% of cells being positive; 1, 0% to 1%; 2, 2% to 10%; 3, 11% to 30%; 4, 31% to 70%; and 5, 71% to 100%. We also rated the staining intensity on a scale of 0 to 3: 0, negative; 1, weak; 2, moderate; and 3, strong. The proportion and intensity scores were then added to obtain a total score (range, 0-8), as described previously (15). Two pathologists who were blinded to the clinical information independently validated the reproducibility of the scores.

### Statistical Analysis

SPSS version 20.0 software (SPSS Inc., Chicago, IL, USA) was used for data analysis. The expression levels of AKT pS473, PCK1 pS90, INSIG1 pS207/INSIG2 pS151, and nuclear SREBP1 in tumor and normal tissues were compared using the independent variable *t* test. The associations between the expression levels of the biomarkers and clinicopathologic characteristics of patients were analyzed using one-way analysis of variance (ANOVA) with the *post hoc* Bonferroni test for multiple comparisons and least significant difference test. The correlations between the expression levels of the biomarkers were analyzed using the Pearson correlation coefficient. Overall survival (OS) was defined as the duration from the date of diagnosis to the date of death or the last known date of follow-up. The survival analyses were performed using K-means cluster analyses to stratify the expression levels of related markers, the Kaplan-Meier method to plot survival curves, the log-rank test to compare survival rate, and a Cox regression model with two-sided Wald tests to calculate hazard ratios (HR) and 95% confidence intervals (CIs). Censored data were used for patients who were alive at last follow-up or who were lost to follow-up. Variables in univariate analysis with *P* values less than 0.05 were included in multivariate analysis. *P* < 0.05 was considered statistically significant. All statistical tests were two-sided.

## Results

### PCK1 Protein Kinase Activity-Mediated Activation of SREBP1 Occurs in NSCLC Cells and Promotes Tumor Growth in Mice

**Figure 1:**
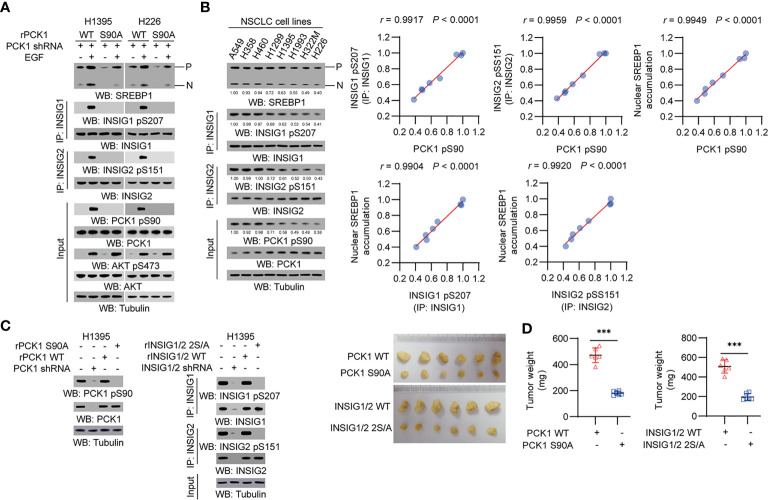
PCK1 protein kinase activity-mediated activation of SREBP1 occurs in NSCLC cells and promotes tumor growth in mice. **(A)** H1395 and H226 cells were transfected with the indicated plasmids. Immunoprecipitation or immunoblotting analyses were performed with the indicated antibodies. **(B)** Immunoprecipitation or immunoblotting analyses were performed with the indicated antibodies in the indicated cells. Correlation analyses were performed. A Pearson correlation test was used (two-tailed) (*n* = 8). **(C, D)** H1395 cells (1 × 10^6^) with expression of rPCK1, rPCK1 (S90A), rINSIG1/INSIG2 or the rINSIG1 S207A/rINSIG2 S151A double mutants were subcutaneously injected into the left or right flanks of athymic nude mice, respectively (*n* = 6 per group). The resulting tumors were resected 28 days after injection **(C)**. The tumors in the mice were weighed **(D)**. Data are mean ± S.D. (*n* = 6). ***P < 0.001 compared with the wild-type group (two-tailed *t*-test). Immunoblotting analyses were performed with the indicated antibodies.

We treated human NSCLC cells with epidermal growth factor (EGF) and found that EGFR activation induced a substantial increase of phosphorylation levels of AKT S473, PCK1 S90, INSIG1 S207 and INSIG2 S151, and the cleavage of SREBP1 in H1395 lung adenocarcinoma (LUAD) cells and H226 lung squamous cell carcinoma (LUSC) cells. Expression of PCK1 S90A mutant inhibited EGF-induced INSIG1/2 phosphorylation and SREBP1 cleavage (**Figure 1A**). In addition, we examined a panel of NSCLC cell lines, including A549, H358, H460, H1299, H1395, H1993, H322M, and H226, and showed that PCK1 pS90, INSIG1 pS207/INSIG2 pS151, and nuclear SREBP1 accumulation were correlated with each other (**Figure 1B**). These results indicated that EGFR activation activates the AKT pS473-PCK1 pS90-INSIG1 pS207/INSIG2 pS151-activated SREBP1 cascade in NSCLC cells.

To determine the role of the signaling axis activation in tumor development, we subcutaneously injected H1395 cells with expression of rPCK1, rPCK1 S90A, or rINSIG1 S207A/rINSIG2 S151A into nude mice. Expression of these mutant proteins substantially inhibited tumor growth in the mice (**Figures 1C, D** **)**. These results indicated that PCK1 protein kinase activity-mediated activation of SREBP1 promotes tumor growth in mice.

### The Expression Levels of AKT pS473, PCK1 pS90, INSIG1 pS207/INSIG2 pS151, and Nuclear SREBP1 Are Upregulated in NSCLC Specimens

**Figure 2:**
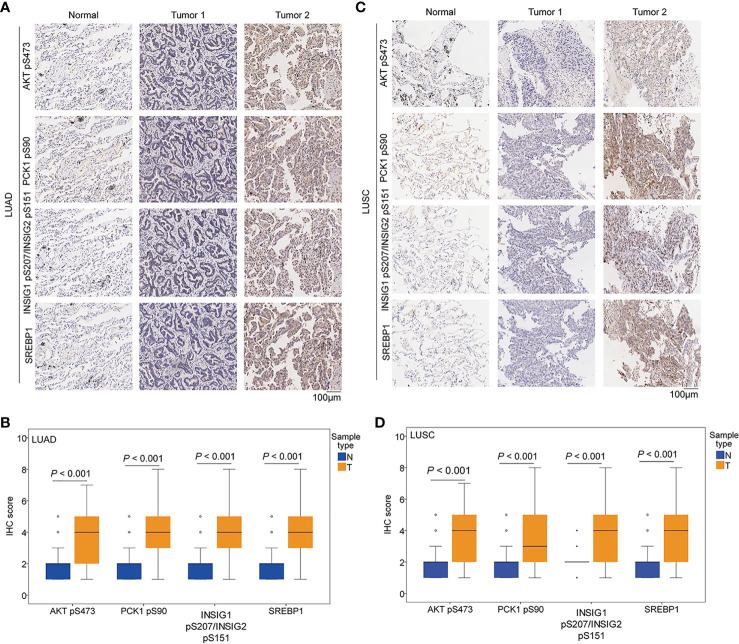
The expression levels of AKT pS473, PCK1 pS90, INSIG1 pS207/INSIG2 pS151, and nuclear SREBP1 were upregulated in NSCLC specimens. IHC staining of 451 paired NSCLC specimens with their adjacent tissues was performed with the indicated specificity-validated antibodies. **(A)** Representative IHC staining of low and high levels of AKT pS473, PCK1 pS90, INSIG1 pS207/INSIG2 pS151, and nuclear SREBP1 expression in LUAD tissues and normal tissues. **(B)** Expression levels of AKT pS473, PCK1 pS90, INSIG1 pS207/INSIG2 pS151, and nuclear SREBP1 in LUAD tissues (n = 306) and normal tissues (n = 302). **(C)** Representative IHC staining of low and high expression levels of AKT pS473, PCK1 pS90, INSIG1 pS207/INSIG2 pS151, and nuclear SREBP1 in LUSC tissues and normal tissues. **(D)** Expression levels of AKT pS473, PCK1 pS90, INSIG1 pS207/INSIG2 pS151, and nuclear SREBP1 in LUSC tissues (n = 145) and normal tissues (n = 145).

We performed immunohistochemical (IHC) staining of NSCLC specimens (*n* = 451), including 306 cases of LUAD and 145 cases of LUSC. Using specificity-validated antibodies (8), we showed that the levels of AKT pS473, PCK1 pS90, INSIG1 pS207/INSIG2 pS151, and nuclear SREBP1 expression were significantly higher in LUAD **(** **Figures 2A, B** **)** and LUSC (**Figures 2C, D** **)** tissues than in adjacent normal tissues, indicating that the levels of AKT pS473, PCK1 pS90, INSIG1 pS207/INSIG2 pS151, and nuclear SREBP1 are significantly increased in NSCLC.

### The Expression Levels of AKT pS473, PCK1 pS90, INSIG1 pS207/INSIG2 pS151, and Nuclear SREBP1 Are Positively Correlated With Each Other in LUAD

**Figure 3:**
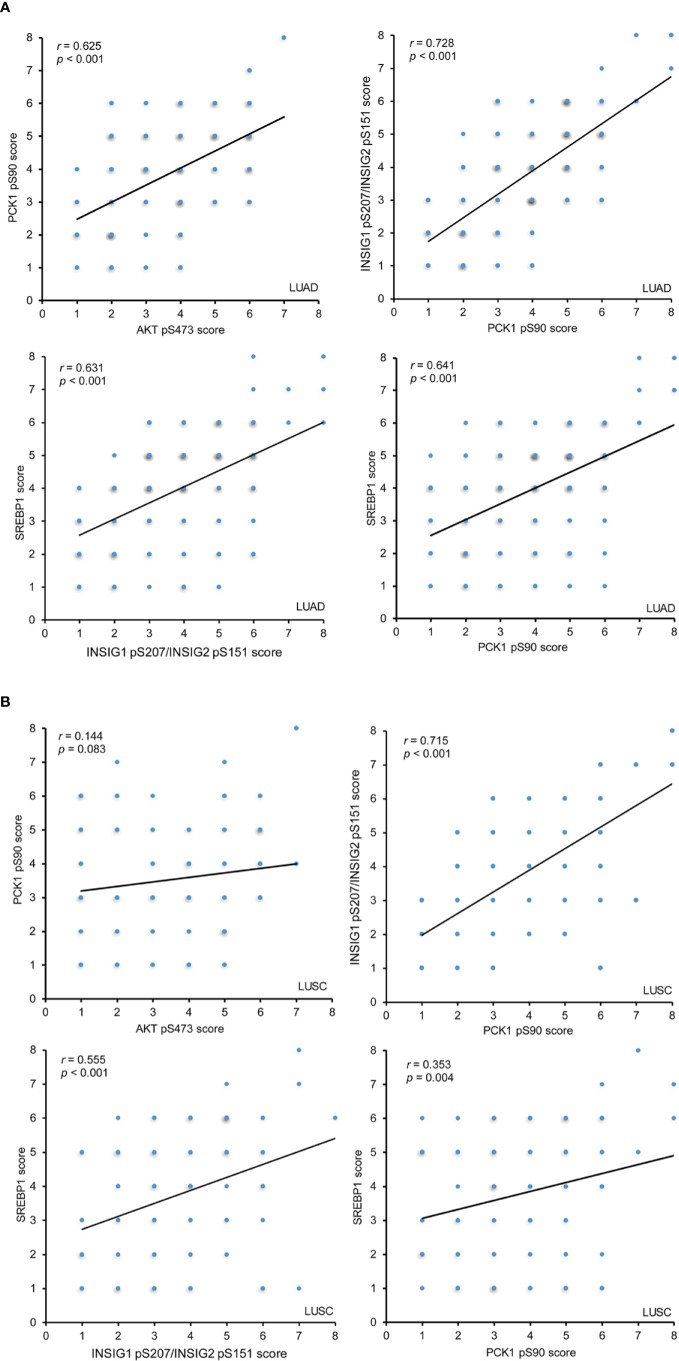
The expression levels of AKT pS473, PCK1 pS90, INSIG1 pS207/INSIG2 pS151, and nuclear SREBP1 are positively correlated with each other in LUAD IHC staining of 451 NSCLC specimens was performed with the indicated specificity-validated antibodies. **(A)** The correlations between the expression levels of AKT pS473, PCK1 pS90, INSIG1 pS207/INSIG2 pS151, and nuclear SREBP1 in LUAD specimens. **(B)** The correlations between the expression levels of AKT pS473, PCK1 pS90, INSIG1 pS207/INSIG2 pS151, and nuclear SREBP1 in LUSC specimens.

To determine whether the AKT-PCK1-INSIG1/2-SREBP cascade is activated in NSCLC tissues, we analyzed the correlation of expression levels of these biomarkers and showed that AKT pS473, PCK1 pS90, INSIG1 pS207/INSIG2 pS151, and nuclear SREBP1 expression levels were positively and significantly correlated with each other in LUAD specimens (**Figure 3A**). In LUSC samples, the correlation of INSIG1 pS207/INSIG2 pS151 with PCK1 pS90 phosphorylation and nuclear SREBP1 expression was strong and significant, whereas PCK1 pS90 levels had a weak correlation with nuclear SREBP1 expression levels and no correlation with AKT pS473 (**Figure 3B**). These results indicated that the levels of AKT pS473, PCK1 pS90, INSIG1 pS207/INSIG2 pS151, and nuclear SREBP1 expression are positively correlated with each other in LUAD specimens and that the correlation of INSIG1 pS207/INSIG2 pS151 with PCK1 pS90 and nuclear SREBP1 expression is significant in LUSC.

### The Levels of PCK1 pS90, INSIG1 pS207/INSIG2 pS151, and nuclear SREBP1 Are Associated With the Progression of NSCLC

To determine whether the activated AKT-PCK1-INSIG1/2-SREBP cascade is associated with the progression of NSCLC, we analyzed the expression levels of these four biomarkers in NSCLC specimens with different T, N, and M (TNM) stages. We demonstrated that all these biomarkers were associated with T stage, N stage, M stage, and TNM stage in LUAD (**Table 1**). However, in LUSC, the expression levels of PCK1 pS90, INSIG1 pS207/INSIG2 pS151, and nuclear SREBP1, but not the expression levels of AKT pS473, were associated with these clinical characteristics (**Table 1**). These results indicated that the expression levels of PCK1 pS90, INSIG1 pS207/INSIG2 pS151, and nuclear SREBP1 are associated with the progression of NSCLC.

**Table 1:** Associations of AKT pS473, PCK1 pS90, INSIG1/2 pS207/S151 and SREBP1 with clinicopathologic features in LUAD and LUSC.

		AKT pS473 IHC score			PCK1 pS90 IHC score			INSIG1 pS207/INSIG2 pS151 IHC score			SREBP1 IHC score		
	Total	Low	High	*p*	Low	High	*p*	Low	High	*p*	Low	High	*p*
**Characteristics (LUAD)**	*n* = 306												
**Gender**				0.659			0.942			0.736			0.637
Male	184	131	53		115	69		115	69		112	72	
Female	122	77	45		71	51		79	43		66	56	
**Age**				0.704			0.489			0.210			0.643
≤60	146	97	49		89	57		90	56		83	63	
>60	160	111	49		97	63		104	56		95	65	
**T stages**				**<0.001**			**<0.001**			**<0.001**			**<0.001**
T1+T2	222	164	58		165	57		57	165		148	74	
T3+T4	84	44	40		21	63		29	55		30	54	
**N stages**				**< 0.001**			**<0.001**			**<0.001**			**<0.001**
N0+N1	219	175	44		168	51		175	44		152	67	
N2+N3	87	33	54		18	69		19	68		26	61	
**M stages**				**<0.001**			**<0.001**			**<0.001**			**<0.001**
M0	298	207	91		186	112		194	104		178	120	
M1	8	1	7		0	8		0	8		0	8	
**TNM stages**				**<0.001**			**<0.001**			**<0.001**			**<0.001**
I+II	176	152	24		162	14		169	7		136	40	
III+IV	130	56	74		24	106		25	105		42	88	
**Characteristics (LUSC)**	*n* = 145												
**Gender**				0.256			0.157			**0.015**			0.534
Male	136	88	48		91	45		89	47		77	59	
Female	9	4	5		7	2		9	0		5	4	
**Age**				0.547			0.866			0.530			0.332
≤60	47	26	21		31	16		30	17		30	17	
>60	98	66	32		67	31		68	30		52	47	
**T stages**				0.906			**<0.001**			**< 0.001**			**0.019**
T1+T2	106	69	37		84	22		84	22		63	43	
T3+T4	39	23	16		14	25		14	25		19	20	
**N stages**				0.060			**<0.001**			**<0.001**			**0.049**
N0+N1	117	82	35		91	26		86	31		70	47	
N2+N3	28	10	18		7	21		12	16		12	16	
**M stages**				0.051			**<0.001**			**<0.001**			**0.022**
M0	142	92	50		98	44		98	44		82	60	
M1	3	0	3		0	3		0	3		0	3	
**TNM stages**				0.218			**<0.001**			**<0.001**			**0.011**
I+II	95	69	26		85	10		81	14		60	35	
III+IV	50	23	27		13	37		17	33		22	28	

PCK1, Phosphoenolpyruvate (PEP) carboxykinase 1; LUAD, Lung adenocarcinoma; LUSC, Lung squamous cell carcinoma; INSIG, Insulin induced gene; IHC, Immunohistochemistry; SREBP1, sterol regulatory element-binding protein 1. Bold values means the comparison results are significant.

### The Levels of PCK1 pS90 and INSIG1 pS207/INSIG2 pS151 Are Positively Correlated With Poor Prognosis of NSCLC Patients

**Figure 4:**
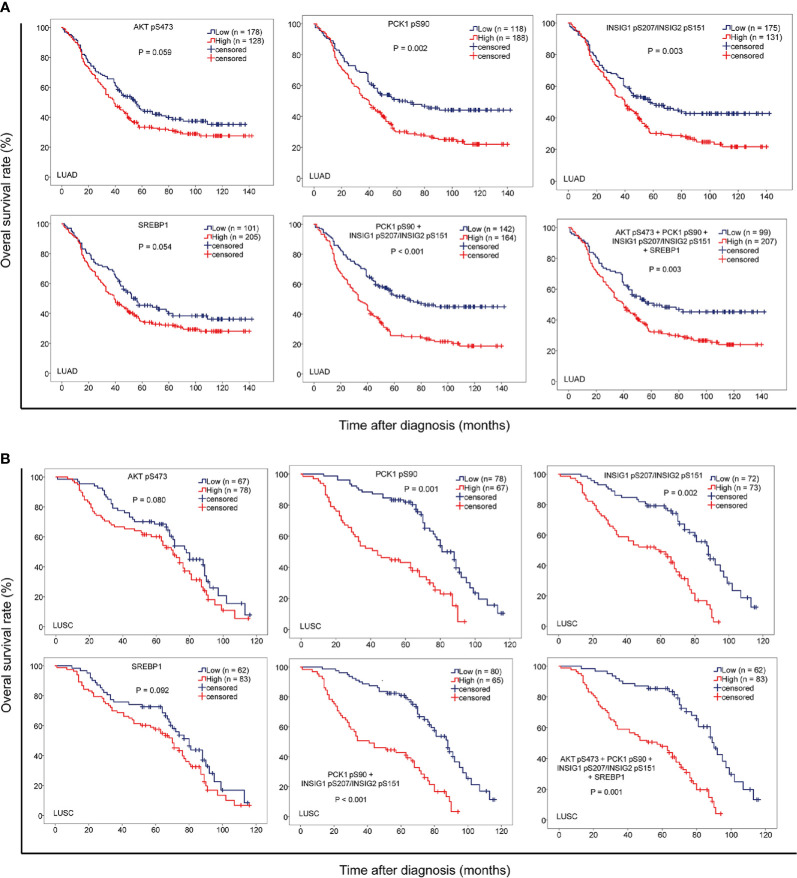
The levels of PCK1 pS90 and INSIG1 pS207/INSIG2 pS151 are strongly correlated with progression and poor prognosis of NSCLC. IHC staining of 451 NSCLC specimens was performed with the indicated specificity-validated antibodies. **(A)** The correlation of the expression levels of AKT pS473, PCK1 pS90, INSIG1 pS207/INSIG2 pS151, and nuclear SREBP1 with prognosis of LUAD patients. **(B)** The correlations of the expression levels of AKT pS473, PCK1 pS90, INSIG1 pS207/INSIG2 pS151, and nuclear SREBP1 with prognosis of LUSC patients.

To further determine the clinical significance of AKT pS473, PCK1 pS90, INSIG1 pS207/INSIG2 pS151, and nuclear SREBP1 expression levels, we determined their relationship with NSCLC patient survival time. We showed that PCK1 pS90 and INSIG1 pS207/INSIG2 pS151 levels, but not AKT pS473 and nuclear SREBP1 expression levels, were positively correlated with poor prognosis in both LUAD (**Figure 4A**) and LUSC (**Figure 4B**) patients. In addition, the combined expression values of PCK1 pS90 and INSIG1 pS207/INSIG2 pS151 and the combined expression values of all four markers were positively correlated with poor prognosis of both LUAD (**Figure 4A**) and LUSC (**Figure 4B**). These results indicated that increased levels of PCK1 pS90 and INSIG1 pS207/INSIG2 pS151 are positively correlated with poor prognosis in NSCLC patients.

### Combined Expression Values of PCK1 pS90 and INSIG1 pS207/INSIG2 pS151 Are an Independent Prognostic Factor for NSCLC

To further determine the prognostic values of PCK1 pS90 and INSIG1 pS207/INSIG2 pS151 in NSCLC, we performed univariate and multivariate Cox regression analyses. Univariate analysis revealed that advanced age (*p* < 0.05), TNM stage (*p* < 0.05), high expression of PCK1 pS90 (*p* < 0.05) or INSIG1 pS207/INSIG2 pS151 (*p* < 0.05), and high combined expression value of PCK1 pS90 and INSIG1 pS207/INSIG2 pS151 (*p* < 0.01) were associated with shorter overall survival time of both LUAD and LUSC (**Table 2**) patients. Of note, in LUAD, combined expression values of PCK1 pS90 and INSIG1 pS207/INSIG2 pS151 were a better prognosis predictor (median survival time, low vs. high: 67.7 vs. 33.1 months; Hazard Ratio (95% confidence interval) (HR (95% CI)), 1.996 (1.496-2.663)) than PCK1 pS90 expression (low vs. high: 67.6 vs. 39.2 months; HR (95% CI), 1.649 (1.223-2.223)), INSIG1 pS207/INSIG2 pS151 expression (low vs. high: 58.2 vs. 40.4 months; HR (95% CI), 1.555 (1.164-2.076)), and combined expression values of the four markers (low vs. high: 62.3 vs. 40.2 months; HR (95% CI), 1.581 (1.185-2.134)). Similarly, in LUSC, combined expression values of PCK1 pS90 and INSIG1 pS207/INSIG2 pS151 were also a better prognosis predictor (low vs. high: 90.2 vs. 41.1 months; HR (95% CI), 3.405 (2.215-5.232)) than PCK1 pS90 expression (low vs. high: 81.4 vs. 44.5 months; HR (95% CI), 2.917 (1.902-4.473)), INSIG1 pS207/INSIG2 pS151 expression (low vs. high: 88.3 vs. 59.8 months; HR (95% CI), 3.129 (2.014-4.861)), and combined expression values of the four markers (low vs. high: 89.1 vs. 57.2 months; HR (95% CI), 3.208 (2.105-4.997)).

**Table 2:** Univariable and multivariable overall survival analysis in patients with LUAD and LUSC.

		Univariable analysis		Multivariable analysis	
	*n*	HR (95% CI)	*p*	HR (95% CI)	*p*
**Characteristics (LUAD)**					
**Gender**					
Male	184	1.000		\	
Female	122	0.826 (0.621-1.098)	0.188	\	\
**Age**					
≤60	146	1.000		1.000	
>60	160	1.665 (0.927-2.354)	**0.035**	1.325 (0.815-1.968)	**0.041**
**TNM stages**					
I+II	176	1.000		1.000	
III+IV	130	2.167 (1.639-2.865)	**<0.001**	1.823 (1.151-2.887)	**0.010**
**PCK1 pS90**					
Low	118	1.000		\	
High	188	1.649 (1.223-2.223)	**0.001**	\	\
**INSIG1 pS207/INSIG2 pS151**					
Low	175	1.000		\	
High	131	1.555 (1.164-2.076)	**0.003**	\	\
**PCK1 pS90+ INSIG1 pS207/INSIG2 pS151**					
Low	142	1.000		1.000	
High	164	1.996 (1.496-2.663)	**<0.001**	1.365 (0.777-2.154)	**0.040**
**Characteristics (LUSC)**					
**Gender**					
Male	136	1.000		\	
Female	9	0.868 (0.418-1.804)	0.703	\	\
**Age**					
≤60	47	1.000		1.000	
>60	98	1.312 (0.895-1.945)	**0.037**	1.215 (0.758-1.856)	**0.043**
**TNM stages**					
I+II	95	1.000		1.000	
III+IV	50	7.493 (4.627-12.132)	**<0.001**	5.630 (3.084-10.277)	**<0.001**
**PCK1 pS90**					
Low		1.000		\	
High		2.917 (1.902-4.473)	**<0.001**	\	\
**INSIG1 pS207/INSIG2 pS151**					
Low		1.000		\	
High		3.129 (2.014-4.861)	**<0.001**	\	\
**PCK1 pS90+ INSIG1 pS207/INSIG2 pS151**					
Low		1.000		1.000	
High		3.405 (2.215-5.232)	**<0.001**	1.443 (0.935-2.156)	**0.033**

PCK1, Phosphoenolpyruvate (PEP) carboxykinase 1; LUAD, Lung adenocarcinoma; LUSC, Lung squamous cell carcinoma; INSIG, Insulin induced gene; IHC, Immunohistochemistry; HR, hazard ratio; 95% CI, 95% confidence interval. Bold values means the comparison results are significant.

Multivariate analysis of patients with NSCLC demonstrated that, in addition to age (*p* < 0.05) and TNM stage (p < 0.05), combined expression values of PCK1 pS90 and INSIG1 pS207/INSIG2 pS151 were an independent poor prognostic factor in LUAD (HR, 1.365; 95% CI, 0.777-2.154; *p* = 0.040) and LUSC (HR, 1.443; 95% CI, 0.935-2.156; *p* = 0.033) (**Table 2**). However, individual PCK1 pS90 or INSIG1 pS207/INSIG2 pS151 level was not an independent prognostic marker for NSCLC (**Table 3**). These results indicated that combined expression values of PCK1 pS90 and INSIG1 pS207/INSIG2 pS151 is an independent prognostic factor for NSCLC patients.

**Table 3:** Multivariable overall survival analysis in patients with LUAD and LUSC.

		Multivariable analysis		Multivariable analysis	
	*n*	HR (95% CI)	*p*	HR (95% CI)	*p*
**Characteristics (LUAD)**					
**Age**					
≤60	146	1.000		1.000	
>60	160	1.375 (0.899-2.102)	**0.040**	1.466 (0.901-2.215)	**0.038**
**TNM stages**					
I+II	176	1.000		1.000	
III+IV	130	1.905 (1.351-2.541)	**0.008**	2.079 (1.498-2.777)	**0.005**
**PCK1 pS90**					
Low	118	1.000			
High	188	0.901 (0.708-1.265)	0.156		
**INSIG1 pS207/INSIG2 pS151**					
Low	175			1.000	
High	131			0.849 (0.568-1.412)	0.375
**Characteristics (LUSC)**					
**Age**					
≤60	47	1.000		1.000	
>60	98	1.333 (0.835-1.988)	**0.041**	1.391 (0.801-2.195)	**0.039**
**TNM stages**					
I+II	95	1.000		1.000	
III+IV	50	6.054 (3.582-10.996)	**<0.001**	6.832 (4.028-11.495)	**<0.001**
**PCK1 pS90**					
Low		1.000			
High		0.938 (0.789-1.352)	0.094		
**INSIG1 pS207/INSIG2 pS151**					
Low				1.000	
High				0.877 (0.622-1.399)	0.232

PCK1, Phosphoenolpyruvate (PEP) carboxykinase 1; LUAD, Lung adenocarcinoma; LUSC, Lung squamous cell carcinoma; INSIG, Insulin induced gene; IHC, Immunohistochemistry; HR, hazard ratio; 95% CI, 95% confidence interval. Bold values means the comparison results are significant.

## Discussion

The abnormal metabolism pathways and gene expression are crucial for tumor cell proliferation. Importantly, these two processes can be mutually regulated (5, 16). In addition to their canonical metabolic functions, metabolic enzymes can perform non-metabolic functions and play critical roles in the regulation of a variety of important cellular activates (5, 6, 17). Metabolic enzymes, such as pyruvate kinase M2 isoform (PKM2), phosphoglycerate Kinase 1 (PGK1), and ketohexokinase (KHK)-A can function as a protein kinase and phosphorylates various protein substrates for promoting tumor development (5, 17–26). We recently demonstrated that PCK1, a gluconeogenesis enzyme, is phosphorylated at S90 by AKT, which is activated by active IGF1R, EGFR, and PDGFR and K-RAS mutations and promotes tumor progression (27, 28). This phosphorylation not only inhibits the canonical enzymatic activity of PCK1 for the production of phosphoenolpyruvate during gluconeogenesis but also translocates PCK1 to the ER, where PCK1 acts as a protein kinase and phosphorylates INSIG1/2. Consequently, nuclear SREBP1 is activated, leading to the promotion of lipogenesis and HCC tumor development (8, 29). In the present report, we reveal the clinical relevance and significance of this signaling cascade in NSCLC.

Gluconeogenesis, the reverse pathway of glycolysis, antagonizes aerobic glycolysis in cancer and suppresses tumor growth (30). In gluconeogenic tissues, such as the liver and kidney, activation of canonical function of PCK1 inhibits glycolysis, thereby tumorigenesis. However, in non-gluconeogenic lung and colon tissues, gluconeogenesis and canonical functions of gluconeogenic enzymes are suppressed, restricting their tumor suppressor functions (30). Importantly, as we demonstrated in our recent publication (8, 29) and current studies, activation of IGF1R in HCC (8, 29) and EGFR in NSCLC inhibited canonical functions of PCK1 and induced PCK1 pS90, INSIG1 pS207/INSIG2 pS151, and nuclear SREBP1 accumulation. In addition, expression of PCK1 S90A and the INSIG1/2 phosphorylation-dead mutant inhibited the growth of tumor in mice derived from human NSCLC cells. These findings suggested that activated AKT signaling in tumor cells converts PCK1 from a gluconeogenic enzyme to a protein kinase that plays a critical role in lipid synthesis.

Our results showed that AKT pS473, PCK1 pS90, INSIG1 pS207/INSIG2 pS151 and nuclear SREBP1 expression levels were positively and significantly correlated with each other in LUAD specimens. While in LUSC, we observed a weak but statistically significant correlation between PCK1 pS90 and nuclear SREBP1 expression levels and no correlation between AKT pS473 and PCK1 pS90. Protein phosphorylation is dynamically regulated by both protein kinases and protein phosphatase. Protein phosphorylation levels and duration can vary among different types of cancer, which have drastic difference in heterogeneity and oncogenic signaling network. Thus, PCK1 pS90 may be differentially regulated in LUSC due to the impact from yet unidentified protein phosphatases, which may result in a rapid turn-over of PCK1 pS90 or AKT pS473 and alter the correlation status between AKT pS473 and PCK1 pS90.

AKT is frequently activated in NSCLC and promotes tumor progression (27, 28). Our work demonstrated that the expression levels of AKT pS473, PCK1 pS90, INSIG1 pS207/INSIG2 pS151, and nuclear SREBP1 were higher in tumor specimens than in their adjacent normal tissues. In addition, the expression levels of these biomarkers were positively correlated with each other in LUAD specimens, and the correlation of INSIG1 pS207/INSIG2 S151 with PCK1 pS90 phosphorylation and nuclear SREBP1 expression was significant in LUSC. Furthermore, the expression levels of PCK1 pS90, INSIG1 pS207/INSIG2 S151, and nuclear SREBP1 are associated with TNM stage and progression in NSCLC. We found that the expression levels of PCK1 pS90 or INSIG1 pS207/INSIG2 pS151 are positively correlated with poor prognosis in NSCLC patients and that the combined expression value of PCK1 pS90 and INSIG1 pS207/INSIG2 pS151 exhibits a better prognostic value than that of each individual expression level. Our results underscore the critical role of PCK1-mediated nuclear SREBP1 activation in NSCLC progression. These findings shed light on the potential for the application of PCK1-mediated INSIG1/2 phosphorylation as a biomarker of NSCLC patient progression and prognosis and highlight the inhibition of the protein kinase activity of PCK1 as a treatment strategy against human NSCLC.

## Data Availability Statement

The original contributions presented in the study are included in the article/supplementary material. Further inquiries can be directed to the corresponding authors.

## Ethics Statement

The studies involving human participants were reviewed and approved by Institute Research Medical Ethics Committee of the Affiliated Hospital of Qingdao University (Qingdao, China) and the National Cancer Center/National Clinical Research Center for Cancer/Cancer Hospital, Chinese Academy of Medical Sciences and Peking Union Medical College (Beijing, China). The patients/participants provided their written informed consent to participate in this study. The animal study was reviewed and approved by Institutional Review Board and the Institutional Animal Care and Use Committee (IACUC) of Qingdao Cancer Institute, China.

## Author Contributions

FS, Conceptualization, Data curation, Formal analysis, Investigation, Methodology, Visualization, Roles/Writing – original draft. XB, Investigation, Software, Data curation. JW, HJ, Validation. GZ, Methodology. LZ, Methodology. DaX, Methodology. SW, Validation. WG, Validation. DoX, Project administration. YG, Project administration, Writing – review and editing. JH, Funding acquisition, Resources, Writing – review and editing. ZL, Conceptualization, Funding acquisition, Project administration, Resources, Supervision, Writing – review and editing. All authors contributed to the article and approved the submitted version.

## Funding

This study was supported by grants from Ministry of Science and Technology of the People’s Republic of China (2020YFA0803300, ZL; 2018YFC1312100, JH), the National Natural Science Foundation of China (82030074, ZL; 81902880, XB), the Beijing Nova Program (Z181100006218032, YG), the Non-profit Central Research Institute Fund of Chinese Academy of Medical Sciences (2018PT32033, YG) and the CAMS Initiative for Innovative Medicine (2017-I2M-1-005, 2017-I2M-2-003, JH), the Zhejiang University Research Fund (188020*194221901/029, ZL), the Leading Innovative and Entrepreneur Team Introduction Program of Zhejiang (2019R01001, ZL), Zhejiang Natural Science Foundation-Key Project (LD21H160003, ZL), Taishan Scholar Foundation of Shandong Province of China (ts20190929), China Postdoctoral Science Foundation (2019M660160, XB), and the Qingdao Postdoctoral Application Research Project (XB). ZL is the Kuancheng Wang Distinguished Chair.

## Conflict of Interest

ZL owns shares in Signalway Biotechnology (Pearland, TX), which supplied rabbit antibodies that recognize PCK1 pS90, INSIG1 pS207 and INSIG2 pS151. ZL’s interest in this company had no bearing on its being chosen to supply these reagents.

The remaining authors declare that the research was conducted in the absence of any commercial or financial relationships that could be construed as a potential conflict of interest.

## References

[1] Bray F Ferlay J Soerjomataram I Siegel RL Torre LA Jemal A. Global cancer statistics 2018: GLOBOCAN estimates of incidence and mortality worldwide for 36 cancers in 185 countries. CA: Cancer J Clin (2018) 68(6):394–424. 10.3322/caac.21492 30207593

[2] Molina JR Yang P Cassivi SD Schild SE Adjei AA. Non-Small Cell Lung Cancer: Epidemiology, Risk Factors, Treatment, and Survivorship. Mayo Clin Proc (2008) 83(5):584–94. 10.4065/83.5.584 PMC2718421 18452692

[3] Liu M Zhang Y Zhang J Cai H Zhang C Yang Z. MicroRNA-1253 suppresses cell proliferation and invasion of non-small-cell lung carcinoma by targeting WNT5A. Cell Death Dis (2018) 9(2):189. 10.1038/s41419-017-0218-x 29415994 PMC5833797

[4] Hanahan D Weinberg RA. Hallmarks of cancer: the next generation. Cell (2011) 144(5):646–74. 10.1016/j.cell.2011.02.013 21376230

[5] Li X Egervari G Wang Y Berger SL Lu Z. Regulation of chromatin and gene expression by metabolic enzymes and metabolites. Nat Rev Mol Cell Biol (2018) 19(9):563–78. 10.1038/s41580-018-0029-7 PMC6907087 29930302

[6] Wang Y Xia Y Lu Z. Metabolic features of cancer cells. Cancer Commun (Lond) (2018) 38(1):65. 10.1186/s40880-018-0335-7 30376896 PMC6235388

[7] Burgess SC He T Yan Z Lindner J Sherry AD Malloy CR. Cytosolic phosphoenolpyruvate carboxykinase does not solely control the rate of hepatic gluconeogenesis in the intact mouse liver. Cell Metab (2007) 5(4):313–20. 10.1016/j.cmet.2007.03.004 PMC2680089 17403375

[8] Xu D Wang Z Xia Y Shao F Xia W Wei Y. The gluconeogenic enzyme PCK1 phosphorylates INSIG1/2 for lipogenesis. Nature (2020) 580(7804):530–5. 10.1038/s41586-020-2183-2 32322062

[9] Jiang H Zhu L Xu D Lu Z. A newly discovered role of metabolic enzyme PCK1 as a protein kinase to promote cancer lipogenesis. Cancer Commun (Lond) (2020) 40(9):389–94. 10.1002/cac2.12084 PMC7494067 32809272

[10] Ji H Ding Z Hawke D Xing D Jiang BH Mills GB. AKT-dependent phosphorylation of Niban regulates nucleophosmin- and MDM2-mediated p53 stability and cell apoptosis. EMBO Rep (2012) 13(6):554–60. 10.1038/embor.2012.53 PMC3367238 22510990

[11] Zheng Y Yang W Aldape K He J Lu Z. Epidermal growth factor (EGF)-enhanced vascular cell adhesion molecule-1 (VCAM-1) expression promotes macrophage and glioblastoma cell interaction and tumor cell invasion. J Biol Chem (2013) 288(44):31488–95. 10.1074/jbc.M113.499020 PMC3814745 24045955

[12] Qian X Li X Shi Z Xia Y Cai Q Xu D. PTEN Suppresses Glycolysis by Dephosphorylating and Inhibiting Autophosphorylated PGK1. Mol Cell (2019) 76(3):516–27 e7. 10.1016/j.molcel.2019.08.006 31492635

[13] Detterbeck FC. The eighth edition TNM stage classification for lung cancer: What does it mean on main street? J Thoracic Cardiovasc Surg (2018) 155(1):356–9. 10.1016/j.jtcvs.2017.08.138 29061464

[14] Lee JH Liu R Li J Wang Y Tan L Li XJ. EGFR-Phosphorylated Platelet Isoform of Phosphofructokinase 1 Promotes PI3K Activation. Mol Cell (2018) 70(2):197–210 e7. 10.1016/j.molcel.2018.03.018 29677490 PMC6114939

[15] Li X Qian X Jiang H Xia Y Zheng Y Li J. Nuclear PGK1 Alleviates ADP-Dependent Inhibition of CDC7 to Promote DNA Replication. Mol Cell (2018) 72(4):650–60 e8. 10.1016/j.molcel.2018.09.007 30392930

[16] Xu D Shao F Bian X Meng Y Liang T Lu Z. The Evolving Landscape of Noncanonical Functions of Metabolic Enzymes in Cancer and Other Pathologies. Cell Metab (2021) 33(1):33–50. 10.1016/j.cmet.2020.12.015 33406403

[17] Lu Z Hunter T. Metabolic Kinases Moonlighting as Protein Kinases Trends in biochemical sciences. Trends Biochem Sci (2018) 43(4):301–10. 10.1016/j.tibs.2018.01.006 PMC5879014 29463470

[18] Yang W Xia Y Hawke D Li X Liang J Xing D. PKM2 phosphorylates histone H3 and promotes gene transcription and tumorigenesis. Cell (2012) 150(4):685–96. 10.1016/j.cell.2012.07.018 PMC3431020 22901803

[19] Yang W Lu Z. Pyruvate kinase M2 at a glance. J Cell Sci (2015) 128(9):1655–60. 10.1242/jcs.166629 PMC4446733 25770102

[20] Li X Qian X Peng LX Jiang Y Hawke DH Zheng Y. A splicing switch from ketohexokinase-C to ketohexokinase-A drives hepatocellular carcinoma formation. Nat Cell Biol (2016) 18(5):561–71. 10.1038/ncb3338 PMC4888794 27088854

[21] Li X Zheng Y Lu Z. PGK1 is a new member of the protein kinome. Cell Cycle (2016) 15(14):1803–4. 10.1080/15384101.2016.1179037 PMC4968903 27105392

[22] Li X Jiang Y Meisenhelder J Yang W Hawke DH Zheng Y. Mitochondria-Translocated PGK1 Functions as a Protein Kinase to Coordinate Glycolysis and the TCA Cycle in Tumorigenesis. Mol Cell (2016) 61(5):705–19. 10.1016/j.molcel.2016.02.009 PMC4888784 26942675

[23] Xu D Li X Shao F Lv G Lv H Lee JH. The protein kinase activity of fructokinase A specifies the antioxidant responses of tumor cells by phosphorylating p62. Sci Adv (2019) 5(4):eaav4570. 10.1126/sciadv.aav4570 31032410 PMC6482012

[24] Qian X Li X Cai Q Zhang C Yu Q Jiang Y. Phosphoglycerate Kinase 1 Phosphorylates Beclin1 to Induce Autophagy. Mol Cell (2017) 65(5):917–31.e6. 10.1016/j.molcel.2017.01.027 PMC5389741 28238651

[25] Jiang Y Li X Yang W Hawke DH Zheng Y Xia Y. PKM2 Regulates Chromosome Segregation and Mitosis Progression of Tumor Cells. Mol Cell (2014) 53(1):75–87. 10.1016/j.molcel.2013.11.001 24316223 PMC3955203

[26] Jiang Y Wang Y Wang T Hawke DH Zheng Y Li X. PKM2 phosphorylates MLC2 and regulates cytokinesis of tumour cells. Nat Commun (2014) 5:5566. 10.1038/ncomms6566 25412762 PMC4259466

[27] Testa JR Bellacosa A. AKT plays a central role in tumorigenesis. Proc Natl Acad Sci U S A (2001) 98(20):10983–5. 10.1073/pnas.211430998 PMC58668 11572954

[28] Tsurutani J Fukuoka J Tsurutani H Shih JH Hewitt SM Travis WD. Evaluation of two phosphorylation sites improves the prognostic significance of Akt activation in non-small-cell lung cancer tumors. J Clin Oncol Off J Am Soc Clin Oncol (2006) 24(2):306–14. 10.1200/JCO.2005.02.4133 16330671

[29] Shao F Bian X Jiang H Zhao G Zhu L Xu D. Association of phosphoenolpyruvate carboxykinase 1 protein kinase activity-dependent sterol regulatory element-binding protein 1 activation with prognosis of oesophageal carcinoma. Eur J Cancer (2021) 142:123–31. 10.1016/j.ejca.2020.09.040 33278777

[30] Wang Z Dong C. Gluconeogenesis in Cancer: Function and Regulation of PEPCK, FBPase, and G6Pase. Trends Cancer (2019) 5(1):30–45. 10.1016/j.trecan.2018.11.003 30616754

